# Hyperloop transportation technologies: practices for open organizing across VUCA contexts

**DOI:** 10.1007/s41469-023-00141-1

**Published:** 2023-04-10

**Authors:** Terri L. Griffith, Ann Majchrzak, Luca Giustiniano

**Affiliations:** 1 Simon Fraser University, Vancouver, Canada; 2 University of Southern California, Los Angeles, USA; 3 Luiss University, Rome, Italy

**Keywords:** Open organizing, Self-organizing, Open innovation, VUCA, Work design

## Abstract

Open organizations are structures in which members of the public engage in work for the organization. Examples include open-source software, Amnesty International, Wikipedia, and Lego communities. Much research focuses on structural design characteristics of open organizations, such as pre-specified task divisions and integration teams. These practices require the organization to a priori structure in response to its mission. Increasingly, however, open organizations like CrowdDoing and Hyperloop Transportation Technologies (HyperloopTT) require public involvement across volatile, uncertain, complex, ambiguous (VUCA) contexts. These open organizations must respond to changing political, competitive, and socio-economic events. Structural clarity is more difficult, and contributors may participate in the creative development of new technologies, new policies, and new sources of funding. Working from practices supporting participant engagement in more stable environments, we qualitatively observe HyperloopTT to understand internal practices for open organizing in more VUCA contexts. We observe four practices allowing for the flexibility, versatility, and accommodations needed for open organizing in such settings. The HyperloopTT practices allow more porosity and self-determination—not simply in how people divide and integrate tasks, but also in the exploration and experimentation of the work itself. More than task workers, we see a new class of open organizing participants: creative work designers.

## Introduction

Open organizing refers to a design in which organizations allow the public to engage in activities traditionally accomplished internally (e.g., von Hippel and von Krogh 2003; Baldwin and Von Hippel 2011; Whittington et al. 2011; West and Bogers 2017; Dobusch et al. 2019). While the open organizing of online knowledge communities receives much attention in the literature (Faraj et al. 2011), previously closed organizations are also opening to participation by the public (Kolbjørnsrud 2018; Radziwon and Bogers 2019). Volatile, uncertain, complex, and ambiguous (VUCA) environments can host open organizing, though we have limited research on internal organizational practices in such settings (Baran and Woznyj 2020). Here, we will contrast stable, often modular, work within open organizations with settings where participants are confronted with volatile, uncertain, complex, or ambiguous contexts.

Many organizations function in one or more VUCA quadrants (Bennett and Lemoine 2014; Burton et al. 2020). However, much open organizing research focuses on cases where work can be predefined with modularized tasks and pre-specified reporting structures (e.g., Puranam et al. 2014; Moffett et al. 2021). We refer to these as stable contexts where the organization can structure in response to its mission: the options are known (Laureiro-Martínez and Brusoni 2018).

In VUCA contexts, the work may not be reducible to clearly defined tasks. It may not be possible to structure clearly toward a specific mission. The options are unknown, and the methods need to be learned or discovered (Simon 1973; Laureiro-Martínez and Brusoni 2018; Cappa et al. 2018; Diriker et al. 2022). As remarked by Burton et al. (2020, p. 1), VUCA landscapes, “design and experimentation are moving ahead as natural *experiments that explore* a portion of the ‘what might be’ space (Burton and Obel 2018)” (*italics ours*). Given that prior research considers modularized tasks and pre-specified reporting structures as practices for open organizing, what practices can support organizations where modularity and structure are less practical given less stability?

Projects such as developing the CERN research detector (Tuertscher et al. 2014), a hyperloop (Majchrzak et al. 2018), or Save Our Ocean’s efforts to reduce ocean plastic (Porter et al. 2020; Diriker et al. 2022) deal with VUCA settings, but they also experiment with open organizing. Consistent across these studies is that participants cannot engage with organizations solely through modularized tasks or predefined reporting structures. The organizations cannot predict the right design given that the solutions are so complex, interdisciplinary, and interdependent with societal conditions (Rittel and Webber 1973; Ferraro et al. 2015). We look to internal practices that allow these organizations to open their participation and remain agile in response to their environments (Baran and Woznyj 2020). We see examples of product and organization designs that cannot be pre-designed by the organizational core but rather “proposed as experiments” (Giustiniano et al. 2019, p. 286) and *impromptu* developed by participants.

At CERN, for example, participants engage in practices of collaborative knowledge sharing and integration within interdisciplinary teams to generate, evaluate, and improve upon multiple novel technical solutions and real-time coordination (Tuertscher et al. 2014). Instead of participants engaging in a specific task, this is reduced from a more extensive set of activities such as debugging a software program or adding a citation to an article. Supporting and maintaining collaborative participant behavior among public participants requires a form of open organizing quite different from the supposition form common among organizations with predefined tasks (e.g., Howison and Crowston 2014; Burton et al. 2017; Dahlander and Piezunka 2020). Nevertheless, much of what is known about specific open organizing practices relies on research on organizations taking on more structured problems (von Hippel and von Krogh 2003; Arazy et al. 2016).

Using a practice lens (Feldman and Orlikowski 2011; Nicolini and Korica 2021), we examine the literature on open organizing and identify four open organizing “everyday” microdynamics (Feldman and Orlikowski 2011, p. 1250). Organizations use these practices with well-structured problems to foster participant engagement and coordination. We see the following relevant to internal practices for open organizing: reporting via a stable structure; identifying with an internally focused, internally bounded organizational identity; sharing information openly; and joining into predefined tasks. We compare these four practices to ones used by an organization working in contexts with VUCA attributes: changing world mobility.

We find substantial differences across the realms of each practice. The stability of the context can explain these differences. Open organizing practices in organizations with more stable contexts encourage participants to become task workers. This aligns with settings where requirements are known. Where participants are confronted with VUCA settings, open organizing practices support participants in becoming designers of their own work. This aligns better to serve innovation through participant creativity and personal growth.

We expand the theoretical boundaries of open organizing in three ways. First, we demonstrate that predefined tasks are unnecessary for open organizing (c.f., Puranam et al. 2014; Bremner and Eisenhardt 2021). More open task alternatives can promote engagement and coordination. Second, we show that open organizing practices should match the context. Finally, we provide a meso link from individual work design practices (e.g., Wrzesniewski and Dutton 2001; Tims et al. 2015) to consideration of organization design and practice as they apply to increasingly popular forms of open organizing.

These contributions build beyond Tuertscher et al. (2014), Majchrzak et al. (2018), Porter et al. (2020), and Diriker et al. (2022) by highlighting more internal organization design practices. Little is known about collaborative, experimental exploration behavior (Majchrzak et al. 2018). Prior work on open organizing in less stable settings has a more macro focus. Diriker et al. (2022), for example, observe that when changes in stakeholders and activities are frequent, alternating closure and openness can, respectively, help to catalyze the contribution across phases and fine-tune the orchestration process. Majchrzak et al.’s designation of HyperloopTT as a “catalyst organization” speaks to differences from collaborative community (Fjeldstad et al. 2012) or similar organization forms previously described as novel (Puranam et al. 2014).

Here we return to our consideration of HyperloopTT with data beyond that available to Majchrzak et al. (2018) and with a focus on internal practices rather than organizational form. We also extend from ideas of closure and openness anticipated by Dobusch et al. (2019) and Diriker et al. (2022): preserving the integrity of strategy development or facing a variety of external relevant stakeholders, respectively. Rather than describe the entirety of HyperloopTT, we explore what takes place at the operational level by considering how practices may differ across stable and VUCA contexts, with a specific focus on open organizing given less predictable, sometimes contentious (Scott and Levitt 2017), interdependences in work.

## Literature review on practices to support participant engagement in open organizing

Openness of an organization refers to the degree to which processes, measures, or actions include external actors (Dobusch et al. 2019). Open organizing involves a fluid interaction between people, technologies, and social action and benefits from a practice-oriented approach (Orlikowski 2000; Feldman and Orlikowski 2011; Nicolini 2012). A practice lens allows us to flexibly consider emergence, improvisation, technology, technology use, and acknowledge a ‘structurational’ process (Orlikowski 2000).

After an extensive review of the literature on open organizing in organizations, we identify four practices to support participant engagement. Given the state of the literature, these practices are drawn from organizations with relatively stable contexts and the possibility of predefined tasks. We have fewer examples of open organizing in contexts prone to VUCA attributes. We can consider the CERN research detector (Tuertscher et al. 2014) and Save Our Oceans (Porter et al. 2020; Diriker et al. 2022). However, these are examples of open organizing emphasizing institutional partners. The concomitant agreements across partners may offer some stability even in their complex, ambiguous, uncertain contexts (e.g., Diriker et al. 2022, p. 11).

### Reporting via a stable structure

‘Onion-shaped’ governance models of accountabilities and responsibilities, with the inner core of the onion holding the highest privileges for problem formulation and decision-making, is a practice researchers often document in open organizations (e.g., Crowston and Howison 2005; Dahlander and Frederiksen 2012; Xu and Alexy 2019; Dionne and Carlile 2019; Giustiniano et al. 2019). These governance models offer increased decision-making responsibility for each layer from the periphery (e.g., Faraj and Johnson 2011; Safadi et al. 2020). The onion structure allows different community members to have differential authority based on their leadership qualities or interests (O’Mahoney 2012), their legal ownership and founding status (Luedicke et al. 2017), and the time and attentional resources they must expend (Faraj et al. 2011). It also allows participants to follow an apprenticeship model for engagement (Lave and Wenger 1991). Newcomers typically start at the periphery of the onion and gradually, by showing increased commitment and capability, move inwards toward a core of decision-makers. Prior research finds onion-shaped structures for participant engagement in such open organizing as OSS projects (Crowston and Howison 2005), Threadless and Local Motors (Langner and Seidel 2015), Lego (El Sawy et al. 2016), and Wikipedia (Joyce et al. 2013; Kane et al. 2014; Arazy et al. 2016).

### Identifying with an internally focused, internally bounded organizational identity

A culture that encourages a strong identity to the organization’s ideology and internal social ties (Langner and Seidel 2015) is another practice to support participant engagement. For example, the ideologies typically focus on the value of open organizing. For OSS and Wikipedia, the production quality is expected to be higher because so many “eyeballs” are involved (Raymond 1999; von Hippel and von Krogh 2003; Stewart and Gosain 2006). This strong cultural identity facilitates participant engagement by motivating those with a similar identity to join and differentiating the organization from outsiders (Stewart and Gosain 2006). The identity is also generally ratified through stories and actions that take on mythical portions (Johnson 2007). Normative behaviors are institutionalized into rules and procedures (Butler et al. 2008). The stronger the identity, the greater the clarity of the boundaries around the community, creating the closure that makes it clear who is in and outside the organization (Faraj et al. 2011).

### Sharing information openly

To support participant engagement, open organizing depends on a principle of transparent information (e.g., Leonardi 2014). The information can include work status, work requirements, feedback on work, access to experts for guidance, and access to instructions. Howison and Crowston’s (2014) analysis of OSS depicts this transparency as embedded in how the platform displays which tasks have been completed, offering direction without human intervention. Faraj et al. (2011) refer to this information transparency as “channeling” participant engagement so that meaningful work is conducted for the organization. Work is known and can be prioritized.

### Joining to predefined tasks

All organizations attempt to match people to tasks (Puranam et al. 2014). In open organizing, this matching is expected to be done by new participants as they join (Faraj et al. 2011; Dahlander and Piezunka 2020). This requires that tasks be predefined so that participants can assess which tasks they are most interested in and can complete in their available time. Von Krogh et al. (2003) refer to this as the ‘joining script’. In OSS, joining scripts typically direct new participants to the code repository for a list of predefined tasks of bugs to fix. Even if the participant has skills to do more than bug fixing, participants are still encouraged to start with bugs to help them learn the work culture of the OSS project. In Wikipedia, the state of the article informs a new participant that work is needed. Specific instructions, such as “citation needed”, direct the participant to predefined tasks (Arazy et al. 2016). In such cases, task interdependence, when reciprocal, appears to be ‘compatible’ (Scott and Levitt 2017). That is, interdependence is addressable via mutual adjustment between tasks of interdependent contributors, achievable via frequent communication and confirmation, and editing and revising outputs.

### Next steps

While the above four practices have value in open organizing where the context is relatively stable, these practices may not apply in organizations dealing with volatility, uncertainty, complexity, or ambiguity. In summary, the research on open organizing identifies essential practices for participant engagement. However, those practices were developed with organizations exposed to stable conditions in which reciprocal task interdependence was compatible: cases where tasks can be broken down and articulated and the co-creation process can be described as continuous editing and revision. We revisit these tasks for work in less stable contexts. In our analysis below, we address this opportunity by examining the practices for participant engagement used by an organization openly organized to tackle the challenge of mass transportation. We leverage a practice lens (Feldman and Orlikowski 2011) because its attentiveness to social and material elements such as artifacts, actions, and interactions is context-specific (Nicolini and Korica 2021). We see the practice lens as a robust approach to understanding VUCA contexts and identifying elements of “designed” or “guided” emergence (Giustiniano et al. 2019; Eisenman et al. 2020).

## Research setting and method

With a focus on open organizing, we conducted a 2-year study of Hyperloop Transportation Technologies (HyperloopTT).^1^ Unlike all competitors in this industry, HyperloopTT founders started the company as an open organization, motivating the public to work part-time in exchange for stock options. They felt the openness would entice highly specialized scientists to offer their expertise. The openness would also help entice part-time efforts from lawyers designing new legal frameworks, marketers preparing slides to convince a skeptical public, business development people dispersed throughout the world convincing funders and governments to consider this new form of transportation, and human resource experts designing new forms of contracts to cover contributors. The openness is marketed broadly and has led to a public following (which eventually included 60,000 social media followers) that often informs company leaders of new business opportunities.

Two of us approached the company founders one year after its inception to discuss the possibility of observing how they engage and support a global set of contributors.^2^ When we started, there was one full-time employee, the two co-founders, and about 20 part-time contributors, primarily in engineering, working on the concept development for the hyperloop in exchange for stock options. By the completion of our 2-year study, HyperloopTT had grown to 800 part-time contributors (vetted with non-disclosure agreements (NDAs) and assigned to projects) and 21 full-time employees on salaries with 39 design patents pending, four patents approved, ten government feasibility studies competitively awarded, the development of a full-scale test track and capsule in Toulouse France (HyperloopTT 2019), and receipt of “investments totaling more than $100 million, including an equity investment of $31.8 million, and total in-kind and land value investments of $77 million” (HyperloopTT 2016).

### Data collection

We obtained our data through repeated conversations with principal participants, shadowing all 80 engineering teleconferences, and reading contributions on the organization’s intranet tools: Facebook’s Workplace, Google Drive, Gmail, Slack, and WhatsApp. Table 1 describes our data collection. The data include 1.27 GB of text history, 7.75 MB of web clips, and over 5500 Workplace posts. Our access allowed us to follow consequential everyday practices (Feldman and Orlikowski 2011) across the organization's development.

**Table 1:** Data collection

Source	Data collection format	Source characteristics (# of interviews in parentheses)	Topics addressed
Co-founders	Semi-structured interviews every 3–6 months—rate determined by level of activity One to five hours for each interview	CEO (6) Chairman (2)	Strategic Vision Updates on business opportunities Reflections outcomes and process Changes to organization since last interview Plans for next steps How they responded to communications on Workplace
Contributors as hyperleaders (hyperleader is HyperloopTT’s term for team leaders)	Semi-structured interviews every 3–5 months—rate determined by level of activity 60 min each	Hyperleaders for Technical Design (29): Engineering Integration (10) Systems Structure (2) Pylon Design (2) Project Management (3) Capsule Design (2) Station Design (3) Power (1) Safety (6) Hyperleaders for Non-Technical Design (28): Marketing & Human Resources (10) Culture (1) Animation (3) Business Development (1) Public relations/Media (1) Global Operations (6) Strategic partnerships (2) Finance (4)	Motivation for and amount of participation Labor arrangements Expectations and areas of disappointments and satisfaction with HyperloopTT Approaches to engagement: technology and personal connections, company outreach Use of external sources of knowledge
Contributors	Semi-structured interviews Thirty to 60 min each Review of approximately 500 online contributions	Ten engineering contributors who had responded to a Workplace *We Need This Work Done* post	Same as for hyperleaders, plus why they responded to a *We Need This Work Done* post in Workplace
HyperloopTT social media followers	Review	Posts made by social media followers to public Facebook account and JumpStartFund.com	Context: reasons for engagement, offers of expertise
Annual company summit in Los Angeles	Notes of all formal presentations and questions to co-founders Informal interactions with attendees	Most attendees were hyperleaders	Same as Contributors
Organizational documents	Review	Onboarding tool HyperloopTT website Jumpstartfund.com NDA Commitment contract Organization chart alternatives	Openness and closedness Structures implemented Changes in information shared Presence of secret workgroups not shown on organization charts
Workplace posts	Review	All log data (about 5,500 posts) from all Workplace groups (secret and open): events, group descriptions, likes, chat messages, comments, replies, and votes Engineering heavily used Workplace, and other groups primarily used it to convey updates to a functional area and the organization overall	Degree of collaborative co-creation occurring in Workplace (vs just using Workplace to make announcements)
Engineering teleconferences	Attended 80 engineering-related meetings, each lasting between 45–120 min	Technical conversations as well as collaboration planning	Same as contributors

### Data analysis

In our in-depth case study (Eisenhardt 1989; Yin 2014), we adopted a circular model of research investigation (Glaser 1965; Flick 2014) informed by our interest and understanding of open organizations. The literature doesn’t expressly speak to what we saw at HyperloopTT. The idea of a practice lens (e.g., Orlikowski and Scott 2014) helped us focus on important characteristics of engagement and performance. Following traditions of interpretive scholarship (e.g., Hatch 1997), we iteratively analyzed the data, alternating between inductive coding to identify possible practices and abductive coding to determine when the initial coding needed to be modified. We were guided by four categories of practices (governance, identity, information sharing, and work design). The categories were sensitizing frames (Gioia et al. 2013), ensuring that the coding was guided but not limited to preconceived definitions of the categories nor the preconceived definitions of practices in the literature.

We iterated between observing a behavior in person at the headquarters/showroom site or online and following up with the actor if it needed to be apparent why the behavior occurred. For example, when one participant asked to start a project, and this request was ignored, we circled back to the Operations Director to understand why. When a participant was reticent to share information with another colleague, we asked in a private follow-up conversation why such information shouldn’t be shared. Throughout this process, the two authors wrote analytical notes in a manner used by interpretive researchers (Nicolini and Korica 2021). They expanded these into memos and PowerPoint presentations made available to the principal participants, who commented on our interpretations of their behaviors. Principal participants included the Operations and Engineering Directors and one of the co-founders. Our intent at this juncture was not to force a pattern of practices but to understand how participants could engage with the organization.

At the end of fieldwork, a third author with no relationship or material interest in the company joined to help avoid interpretation biases in the analysis (Eisenhardt 1989; Bartunek and Louis 1996). The third author interrogated the memos, asking us to work back and forth between memos and original field notes (Nicolini and Korica 2021). We coded all the data with respect to each of the four categories of practices rather than the specific practice. For example, all behaviors we observed related to information-sharing were coded into the category of information-sharing practices, not the specific practice of open information-sharing.

Our next step was to examine, within each category, all the behaviors that exemplified this category. We looked for patterns within the category. This iterative process included the third author, principal participants, fieldwork notes, and analytic memos. New memos and PowerPoint presentations were prepared for the principal agents to obtain feedback on our pattern analysis in labeling and describing practices. As such, the findings below have passed an essential validation of ‘member checking’ (Nag et al. 2007). At no point were these practices formalized by the organization in internal documents. Instead, we adhered to a practice lens in which social practices are foregrounded, “defined as routinized regimes of materially mediated doings, sayings, knowing, and ways of relating that form the building blocks for understanding organizational phenomena” (Nicolini and Korica 2021 p. 1277).

## Practices for open organizing in less stable contexts

Our analysis uncovered that contributors were exposed to the following VUCA circumstances: *volatility* of global and regional economies affecting the development of transit and local volatility in terms of team membership*, uncertainty* related to design and regulation fluctuations, *complexity* in managing global contributions via crowdsourced teams distributed across countries, and *ambiguity* related to the development of a novel transport solution facing reciprocal contentious interdependences.

Below we identify four practices paralleling but different from those developed across organizations with more stable contexts. To preserve anonymity, we disguise the roles of individuals. In the discussion, we suggest that these practices can be further abstracted. These practices engage participants as designers of their own work to better serve their creativity and personal growth needs. The new practices are substantively different from practices that engage and manage participants as task workers in stable contexts.

### Practice 1: reporting via a dynamic porous structure

**Fig. 1:**
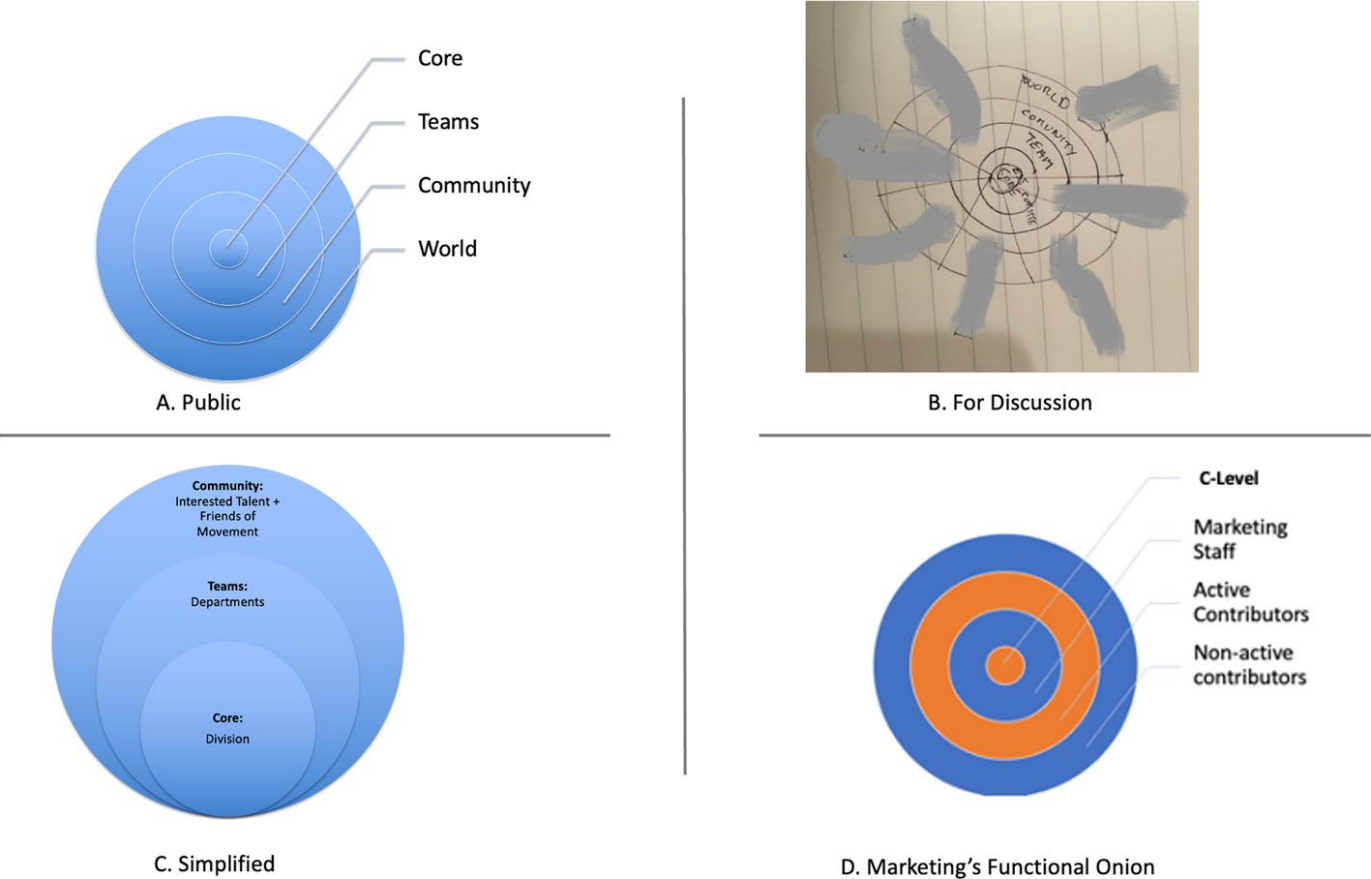
Different presentations of onion structure

HyperloopTT senior leadership repeatedly clarified that when communicating to external investors, the governance structure was highly formalized and quite traditional with the core committed contributors in the center of the onion structure and either the “world” or less active contributors on the periphery. They shared three versions of their formalized governance structure with us: see Fig. 1A, C and D.

However, attending meetings, conducting interviews, and reviewing all Workplace posts made us realize that the public versions differed from an internal view of the onion structure. Figure 1B shows a hand-written governance structure, indicating that the circles were far more porous than the other figures would infer. Another contributor, when commenting about the formalized onion structure:

This is fine as a marketing and branding tool; quite good actually. It just doesn’t tell us how work is done, which is typically a purpose of an org chart (Fig. 1D).

In attempting to understand how work is done through the reporting structure, we identified not four circles but seven, as described in Table 2.

**Table 2:** The HyperloopTT onion circles and commitment mechanisms

Onion circle	Representative titles and numbers	Commitment mechanisms	Examples of actions taken within circle
Strategic decision-making circle	Founders: CEO, COO CFO, Global Operations Marketing Lead Engineering Lead	Stock options, Independent Contributor Agreements (ICA)/Non-Disclosure Agreements (NDA), a few with employee agreements. All working full-time	Pursuit of a feasibility study, presentations to investors, interviews with media, meetings with large potential corporate partners
Hyperleaders	55 hyperleaders (team leaders) working 10 to 40 h a week	Stock options, ICA/NDA, few with employee agreements. Most part-time	Recruited individual and corporate contributors, weekly meetings with team members, created sprints around deliverables, obtained tool resources to do work, identified blockers, and discussed with other hyperleaders to remove blockers. Ex: The Finite Element Analyst Hyperleader was responsible for modeling the airflow around the tube; the Station Team Hyperleader was responsible for designing the stations where hyperloops would dock; the Safety Team Hyperleader was responsible for creating safety regulations that aligned with various country regs; the Optimization Team Hyperleader was responsible for creating maps of routes for the hyperloop. Broadly, managing the most interdependent work within and across teams
Vetted team contributors	Hundreds of crowdsourced contributors assigned to one of 55 teams. Active members ranged from 240–500 at any one time. Titles included: Operations Liaison, Propulsion Engineer, Agile Work Process Consultant, Marketing Analyst, Engineering Support, Communications Engineer	Stock options, ICA/NDA No one worked full-time	Team membership gained through invitation or individual request and tracked by membership in related Workplace Groups. Included some employees of external partners (see below) working alongside contributors. Since they were contributors and not employees, they could not be “assigned” tasks, but they could suggest tasks and then volunteer to carry out the tasks in exchange for stock options. Example interdependent tasks included creating marketing videos, designing the pod, developing the carbon fiber material used on the pod, developing the vacuum pump technology, designing the brake system, designing the station, administering the coordination tool, Workplace
Vetted non-team contributors	Those not assigned to teams. Contributors may have moved from a team or vetted with the expectations that they would do “*some good*”	Stock options, ICA/NDA	Independent work: designing a database of marketing information, designing an agile scrum process for the teams, writing AI algorithms to automate time reporting
External partners	40–50 companies	Contracts, largely focused on stock options rather than cash	Contributing employees of partner companies engaged in either individual-level work or teams (blended with contributors). For example, a partnership with PriestmanGoode and Carbures created the capsule. An entire team at Carbures worked on the capsule partnered with two engineering designers at HyperloopTT
Friends	60,000 unassigned crowd-members	Largely connected through websites set up by HyperloopTT on Facebook and Twitter. Number determined by follower count	Crowd-members offered business opportunities to HyperloopTT by setting up meetings with government officials, identifying potential partnerships with companies and governments. The Toulouse Innovation Centre, a collaboration with the Toulouse government, came about this way. Similarly, for a meeting with Putin and the Slovakia partnership. Group in India autonomously organized a conference to highlight the company and invite the founders to talk at the conference, individuals contacting the CEO through LinkedIn to discuss the possibility of insuring a hyperloop
“The World”	Company materials described this outer circle of the onion as offering “*potential sphere[s] of influence and future talent*”	None	Anyone who might potentially contribute, ride, or be influenced by the availability of the hyperloop in the future – literally, the world

Table 2 shows each circle's titles, commitment mechanisms, and sample activities. Our examination of the activities indicates that contributors closer to the center, such as hyperleaders, were part-time, compensated the same as those further from the center, and did not necessarily provide more time. In our first meeting with the founder and CEO, he highlighted that he intended the onion *“to be porous*” so that those who have time when their skills are needed most could take over more control. Then when their skills are no longer needed, or their regular jobs keep them from taking control, they move away from the center. Another contributor spoke about the circles as *“cellular membranes”* that people should feel free to cross toward and away from the center. For example, one of the members of a scientific council of advisors (vetted non-team contributor circle) met every three months to advise the Engineering Integration Lead (vetted team contributor circle). He also served as a specialist on gravity’s effect on passengers and joined the gravity team (vetted team contributor circle). That same individual also served as a Business Opportunity Specialist (vetted non-team contributor circle), working to find AI experts within the partner circle. Thus, this single contributor simultaneously played roles in three of the seven circles of the onion.

The reporting structure was dynamic in the sense that it was problem-driven rather than organization-driven, as illustrated in this quote from another contributor:

*…our organizational structure operates like a balanced functional matrix (perhaps with a little more variability among individual teams). Our organization is broken up into task-based team systems, but we don’t necessarily subscribe to hard top-down managerial styles—so it’s more problem-oriented architecture. The power within the organization is more diffuse and flexible depending on the nature of the task being solved. And it seems that the individual teams determine the extent of the cross-collaboration between other teams.*

The reporting structure supported this dynamism by making the distinctions between the circles by the degree of interdependencies among multiple specialty areas, rather than specific tasks. One contributor noted that “*innovation occurs BETWEEN departments.*” The reporting structure supported interdependent action when required and independent action when possible. Thus, when contributors conducted a feasibility study, there was no hyperleader per se. Instead, several interdependent contributors were getting the work done together. In some sense, the reporting structure came and went. There was a circle for those preferring independent work, such as a contributor in Mongolia who independently wrote algorithms to automate time reporting. An Operation Team member described the structure as *“like an accordion; [a] dynamic of growing, releasing, parking into special projects.”*

Finally, the contributors maintained this dynamism as they discussed and acted on ways to improve contributor engagement. A briefing labeled “The Functional Onion” described a problem of engaging those preferring independent work into the more prevalent interdependent work incumbent in an engineering research program. One contributor commented:

*[We need to do a better job of]… informing/communicating [to contributors] to bridge between active projects, foreshadow future potential projects, cross-share project experience with each other and Hyper[leaders] more broadly, [and] create community.*

This concern led to the piloting of several alternative reporting structures, including one in which everyone must report to a hyperleader, another in which projects can be spun off and self-organized, and another in which the vetted contributors not yet on a team are moved to a talent pool, drawn upon when needed. “*The way we are working is different and not always everything works as well as we would like to, but overall we are making enormous progress.”*

### Practice 2: identifying with an external movement that prioritizes societal change with little regard for organizational boundaries

**Fig. 2:**
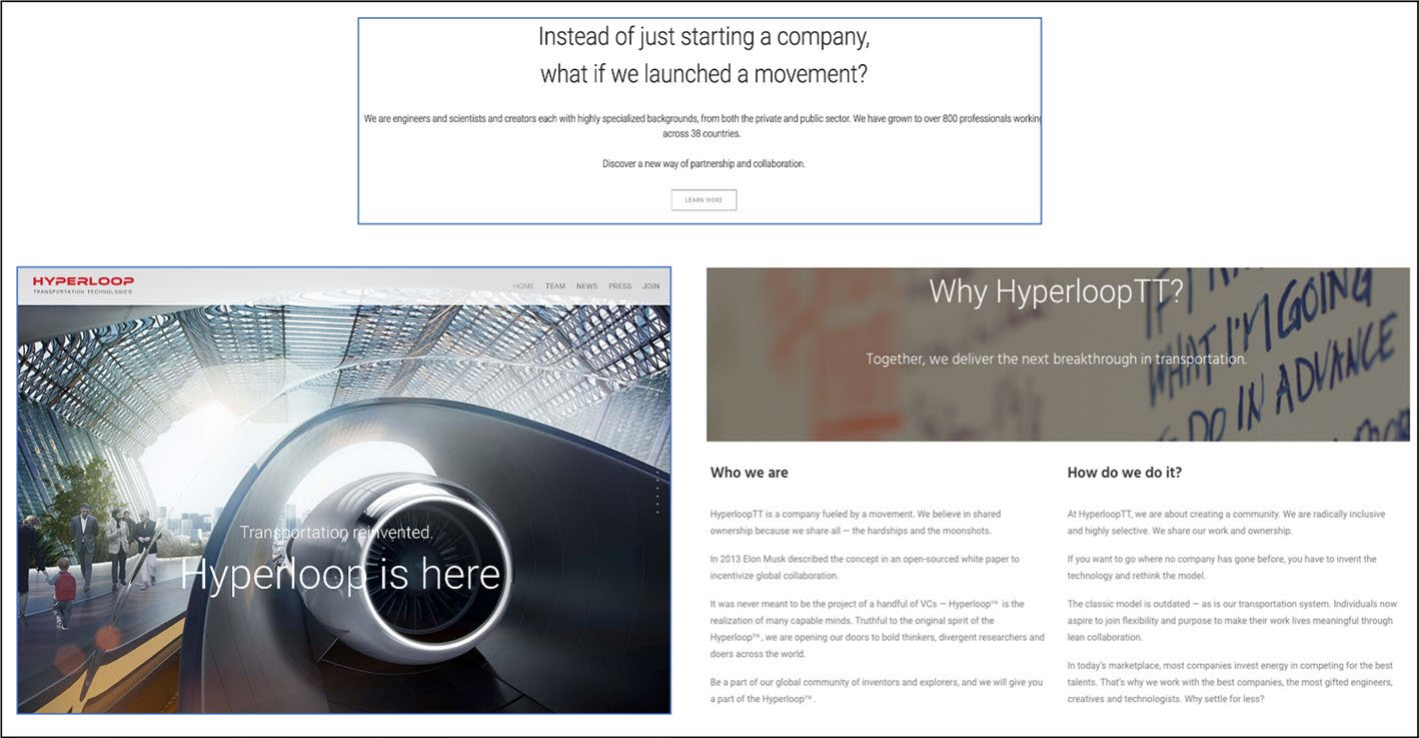
Examples from HyperloopTT website at time of data collection

Figure 2 shows screenshots from HyperloopTT’s website. These emphasize the social movement nature of the organization: “*What if we launched a movement?*” “*HyperloopTT is a company fueled by a movement.*” A review of speeches by the co-founders highlights the social change envisioned: “*replacing cars and trucks for long distance transport, moving population density away from cities*.”

The hyperloop is seen as clean energy compared to the railways, cars, trucks, buses, and airplanes it competes against. But accepting a hyperloop future requires substantial social change. Here are some of the issues raised in internal documents: the development of a hyperloop requires complex regulatory change (as a train? plane?), volatile shifts in social pressure for reducing carbon emissions, uncertain government allocation of infrastructure funds, insurance industry adoption of new safety standards, designs to assure that time in a tube isn’t claustrophobic, and the necessity of an ecosystem of shuttles to ensure the last mile of transport does not overcome the benefits of the carbon–neutral hyperloop. Early interviews with the co-founders indicated that they did not know where the first hyperloop would be built or when; it didn’t matter, they said, because eventually, it would happen with enough social pressure. So, they didn’t just want anyone to do tasks for HyperloopTT; they wanted *“engineers and scientists and creators with highly specialized backgrounds”* who wanted to be part of a *“global community of inventors and explorers.”* They called themselves *“radically inclusive”,* eventually having contributors across 36 countries.

One co-founder repeated this focus on a movement in much of their communication externally and with us: “*Our company has made clear from the beginning that we believe the hyperloop itself is a movement and we are participants within that movement.”*

Being part of a movement for the co-founder meant that the organization and the contributors should be willing to partner with anyone, including competitors or even Elon Musk. Thus, the boundaries of where HyperloopTT as an organization exists and all the movement’s actors are intentionally fuzzy. A collaborative document where people brainstormed about how to get work done opened with, *“Unique to our approach is that the work is crowdsourced enabling us to draw on the talents of a far larger group than if we were a bounded, traditional, organization*.” In the words of the founder*:*

*We partner with numerous specialized organizations to piece together some of the more technical/specialized aspects of the hyperloop. We work with universities and research organizations on experimentation. We also work with governments on the fiscal and policy implementation. These aspects make the hyperloop itself more like a platform than a one-sided market. The network effects that we derive come from the collaboration of the parties and through that collaboration we cultivate the industry stakeholders. The more open we maintain the system, the greater the breadth of the eventual stakeholders.*

When competitors announced the achievement of significant milestones, senior leaders wrote on Workplace to remind contributors that competitors’ successes were everyone’s success since the goal here was to change transportation:

*All- we’ll see news today that Hyperloop One announced a private test of their system. This validates that [h]yperloop is here, it’s doable, and achieving levitation in a vacuum tube is not that difficult. Everyone in this space shares a common goal to bring this to life. And a rising tide floats all boats. Let’s stop for a second and as fans of [h]yperloop* *positively recognize their efforts...and then get back to building something amazing….We’re not here to just develop pipes and pods. This movement is much more, and this is one example of many things we’ll be able to accomplish.*

Contributors resonate with this broader, externally focused movement. Here are example quotes from contributors when asked what they identified within the company:

- *Sure it would be nice if we came out on top and I was able to buy an island with my stock options, but as long as we’ve changed the world, that’s what counts. Besides it will be a big market when it finally catches on.*
- *It’s a different way to build a business. Because we’re not a company, we’re a movement. And it’s the passion and talent from this group that has made this possible.*
- *Impossible enough to be possible*.
- *The eyes are afraid, but the hands are doing*.

**Fig. 3:**
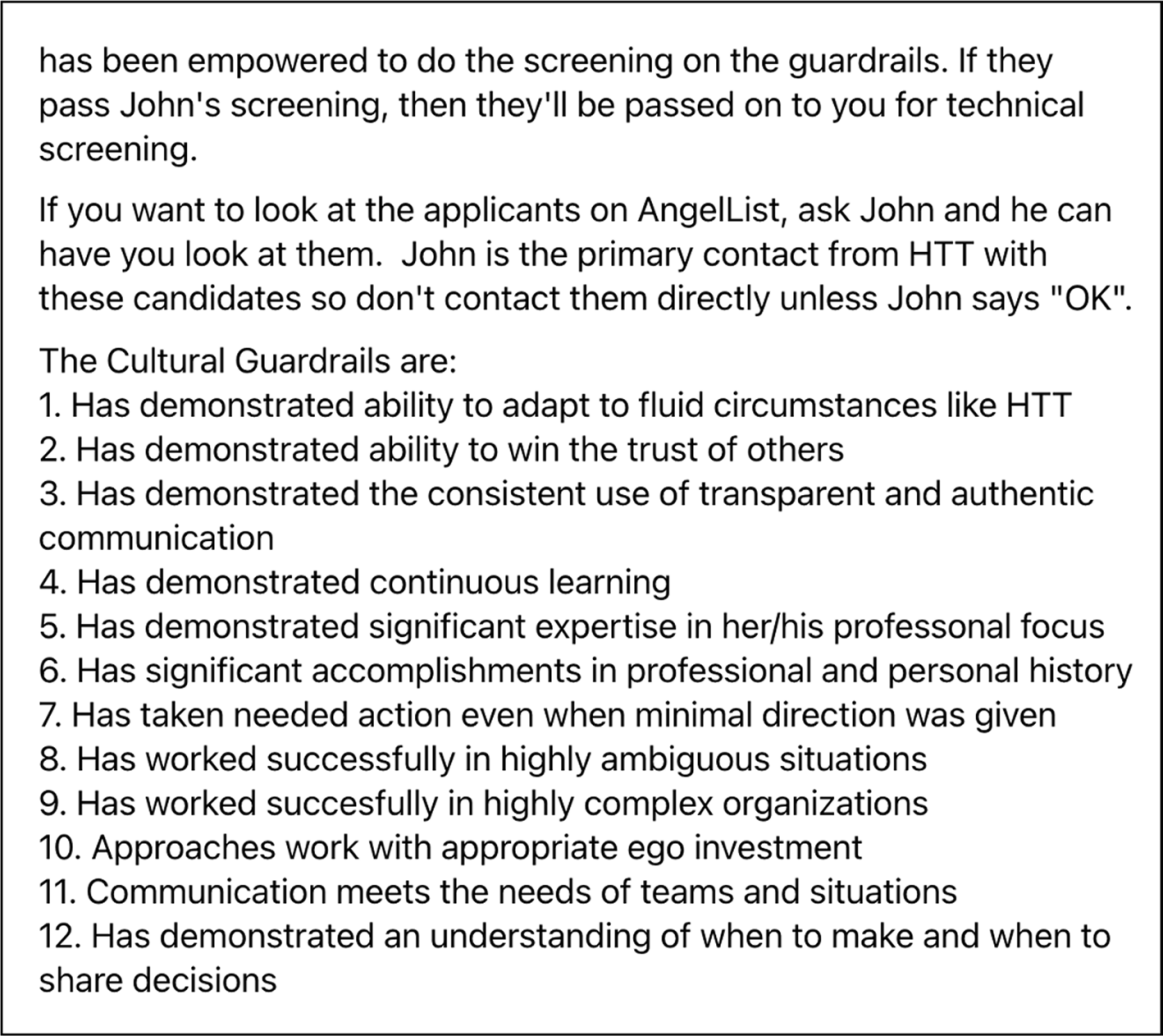
Screenshot from Workplace post on cultural guardrails

Contributors built tools and practices to support this identification. As onboarding increased, a small team created a document, “Cultural Guardrails”. These guardrails, as shown in a screenshot in Fig. 3, indicate that contributors need to have the movement paramount and then adapt to fluid, ambiguous, complex circumstances with minimal direction.

Moreover, when contributors completed the onboarding process consisting of an NDA and signing a contributor agreement indicating a willingness to contribute a certain number of hours in exchange for stock options, the contributor was again met with a movement-focused congratulatory note:

*Congratulations! You have been officially selected to be a member of the Hyperloop movement and we’re thrilled to have you on board!*

*Our cities are polluted, our roads are crowded, and our travel experiences are now defined by chaos and calamity. Hyperloop is not just about record-breaking speed, it’s about improving the entire transportation infrastructure. It’s time to use resources smartly, travel green, and lower the cost of mass transit for everyone.*

*History will know your name.*

*Keep on disrupting!*

Additional written materials pointed to the broad mission understanding. For example, during a discussion of how to describe to others the way of working at HyperloopTT, a contributor responded:

*The first rule of branding is that you never tell people you’re different, special, visionary, smart, etc.—rather, you tell people what you have accomplished and let them come to those conclusions themselves, because that’s how the terms become credible. So in this case, for example, we point to the specific companies that have been inspired by us to transform the way they get work done. We tell the stories of what exactly they learned from our organization and applied to theirs and what the results have been. And then other people say, “Wow, you’re kind of leading a movement there”—which becomes believable because they’ve been shown instead of told.*

There were also more public versions. Though a global organization, HyperloopTT has had a Los Angeles showroom housed in a hangar once used by Howard Hughes’ aircraft company from the earliest days. Just as you enter, a glass-walled conference room became home to visiting contributors’ multilingual wall graffiti. Some of the inscriptions are personal: “*As long as it’s fun”,* or inspirational, “*CAMO HAПPEД” [ONLY FORWARD].* Others focus on the broader perspective: “*Making the future of transportation reality*”, “*Let’s innovate—No Boundaries*”, “*I’m interested in making future* [sic] *and it levitates*.”

In sum, there is repetition and communal support for the external movement of both creating a hyperloop and a “radically inclusive” organization. The precise role of HyperloopTT in the movement is of less concern.

### Practice 3: designing information sharing

*As members come and go and also the fact that they are continuously on and off from [HyperloopTT] because of their main job... Their memory works differently... they need to invest time to catch up every time, to get back on track, to remember what were the main issues, who was doing what... It probably requires a new level of skill sets: for example being able to execute and perform with a lower level of information, develop intuition on what could work, learn how to align fast with your colleagues, etc.*

We read this as contributors are expected to deal with ambiguity, complexity, and volatility. Senior leaders had many discussions with the authors about information-sharing related to these issues. They were worried that contributors would take too much time from others by asking for background information rather than getting work done. Since stock options were allocated by time, and time, in general, was limited, senior leaders didn’t want contributors to spend all their time getting caught up. They were also worried about intellectual property (IP) theft. Senior leaders initially indicated that they solved the information-sharing problem by not openly sharing all information. Instead, they “*compartmentalize the information-sharing; nobody has access to information they don’t need*.”

Yet “*many contributors believe they should be allowed to know everything; passionate people want to know everything.*” Consider this exchange:

*Lead: I think we need to split the info up in areas. This is a tricky part.*

*Contributor: The more you split the information up, the more fragmented everyone’s work [is] which helps IP but hurts cross-team coordination and communication. There must be an intermediate route.*

We explored how the company managed this tension between contributors who wanted to resolve ambiguity and uncertainty and senior leaders who were worried about information-sharing. Two patterns of non-open information-sharing practices surfaced: (1) ‘triangulated permissioning’ before sharing information and (2) blocking awareness of some work activities from most contributors in what we call ‘secret gardens’.

Senior leadership did not know where the IP would be created, so they were against creating a specific IP structure. Instead, they wanted the contributors to accept responsibility for IP creation and theft prevention personally. In the words of a founder speaking to an individual contributor: “*Be open [to] IP and design a structure that can make you know enough to work, but not too much to steal it or bring it to somewhere else… we rely on your own motivation, trust, to allow us to achieve this.”*

However, this simple directive took much work to implement. Contributors were often not informed about the current state of negotiations with suppliers, customers, and partners. Contributors repeatedly complained about needing to know if information they were sharing with others in the company was confidential or not. What emerged was ‘triangulated permissioning’. If at least two contributors (outside of those personally involved in creating the information) knew of the information, the information was considered ‘available enough’ for other contributors to feel comfortable sharing it in the company. If I know A, and you know A, then I can probably share A with a third contributor. If they were unsure if other contributors knew about the information (based on reading Workplace, by reading the distribution lists of recipients on an email, or attending a teleconference where others failed to mention the information even though relevant), the contributors would not share the information. Instead, they would ask the source known to have generated the information for permission to share. This practice was quite clever since the confidentiality of the information is likely only to be known by specialists familiar with the technology and the state of the industry; by asking the information source for permission to share, contributors are not inadvertently passing on confidential information.

The ‘secret gardens’ are the second definition pattern for information-sharing: teams seeming to disappear and reappear from organization charts, report-outs at meetings, and discussions about progress.

We saw this first when we discovered through interviews the existence of an innovation lab that only a few engineers knew about. We asked the founder why there was no public mention of the lab (such as in Workplace), and he said that, by surfacing the innovation lab, he would have to field more questions [taking his time and that of the contributors]. There were many additional examples where groups appeared, disappeared, and reappeared. For example, a group formed to solve one of the physics problems challenging the hyperloop design. The group tried several different approaches, but none seemed to succeed. One day, a lead reorganized the group around new contributors explicitly recruited to focus on the problem. The lead told the group not to talk to others while working out their solutions. The group essentially disappeared from discussions, from Workplace, and updates. The group resurfaced only when they’d developed a solution, “*ensuring that the project, critical for IP, is not leaked.”* Because the secret teams were unknown, engineers outside the secret team could proceed without trying to synchronize their work with the secret teams, freeing them up to develop bold solutions.

### Practice 4: leaving work intentionally undefined

*“Thought of a job is out of place here, don’t want to even say ‘job’ anymore. Totally different workforce*.”

The concept behind the work design was originally to have sprints, following the agile SCRUM methodology of software development. Small teams of specialists would come together to solve a specific problem, solve the problem, and then disband to join the next team. This didn’t work as well as anticipated because it was challenging to match the problem needs with the specialists available at the moment. The operations director was loathe to publish a list of these problems (to market specific needs) because he didn’t want competitors to know which problems were as yet unsolved.

Given this ambiguity, contributors shifted their work in ways other than simply responding to a list of predefined problems. A team created a custom Agile document entitled *HyperSpirit Process Guide.* The first listed step: “Self-Organize”. The practice evolved to include ad hoc sprints rather than planned sprints, but only when there was a problem and specialist skill match. For example, when the right combination of a doctor familiar with weightlessness issues on nausea and a physicist knowledgeable about weightlessness became known to each other, they worked together on the issue of creating a design to minimize passenger discomfort.

Second, to compensate for contributor specialists having limited time, a specialist might partner with another contributor to respond to uncertain availability. Since every contributor had their own time limitations, there were cases where some highly specialized contributors could not spend needed time engaged in the interdependent discussions related to the problem. What emerged were ‘go-between buddies’. For example, the go-between buddy attended meetings, took detailed notes about the discussion, and then connected the specialist with this information so that the specialist could independently continue to try to solve the problem. The go-between buddy would then share the draft solution with those interdependently related to the solution. The buddy would again take notes and then meet with the specialist. This iterative process continued until the problem was solved.

A third way to continue work without predefining it was that hyperleaders encouraged specialists to work on a problem, not necessarily to solve it. Some problems were too complex to solve in the short time the contributor might have. Instead, contributors worked on the problem via additional experimentation to take the task to the next level of exploration. Then they passed the work’s current state to the next specialist. A senior leader described contributions as dominos:

*It’s a small block, by falling down it can move something twice its weight. So imagine our little domino. Every single action we do in our crowd affects [the result]. Your little action is allowing us to build the hyperloop.*

Finally, work was left undefined to encourage projects to start up and see if there was sufficient energy and focus on continuing the project. By the end of our observation period, there were 150 simultaneously executed projects among contributors. For example, a group created a ‘*crowd council*’ and went so far as to recruit external experts to support the continued development of the crowdsourced approach. The projects only continued to the extent that there was contributor enthusiasm. In the case of the crowd council, after an initial start-up, the project fizzled when the director of HR left.

These practice patterns of leaving work undefined were not without challenges. The most critical challenge was that the part-time contributor had another full-time job. Deadlines could be missed. Hiring full-time employees responsible for the deadlines and supporting the specialists was the ultimate response. In this way, according to an employee:

We have the opportunity to work with so many people who are passionate. Tons of enthusiasm. [I just have to make sure I can cope if a specialist] may have to leave in the middle of a creative sprint. We are not there to optimize [a single contributor’s work], but to catalyze.

## Discussion

Contemporary organizations increasingly address grand challenges (e.g., Tuertscher et al. 2014; von Hippel and von Krogh 2016; Porter et al. 2020; Diriker et al. 2022) by adopting aspects of open organization, even when the contexts have volatile, uncertain, complex, and ambiguous attributes. VUCA can also affect more traditional organizations exposed to global societal phenomena: the global spread of COVID-19 is an example. The COVID-19 pandemic demonstrates that handling VUCA contexts with solutions crafted as if they were for more stable contexts generates new and more significant issues—i.e., adopting old routines in the face of unseen public health issues.

More than the openness to the crowd, less stable environments challenge the traditional pillar of activity coordination and organization design in general. Many open organizations can benefit from public crowd contributions (Majchrzak and Malhotra 2020). The benefits accrue if the organizations deal with well-structured problems (e.g., Lego Ideas contests) and, therefore, can assign defined tasks (e.g., design a space-themed set with 150–250 bricks) beyond their organizational boundaries (c.f., Garud et al. 2006). This background applies to both crowd-open (e.g., Lego) and crowd-based (e.g., Wikipedia) forms of organizing (Giustiniano et al. 2019).

However, open organizations taking on complex work in VUCA contexts must manage complexity and interdependencies among tasks that may unfold as contentious. That is, “for which the interdependent parties have one or more conflicting subgoals” (Scott and Levitt 2017 p.97). In such cases, any attempt to turn ill-structured problems into well-structured ones may trigger additional complexity. At the beginning of its journey, HyperloopTT started with the idea that the organization structure could match the project’s main components (see sources in Table 1). In other words, they assumed that tasks were finite and definable with components that predictable interdependencies could link. Soon they realized that problem formulation and solving were unfolding in parallel, and the tasks were often complex and ambiguous. At the same time, contentious interdependence among work components fostered the necessity to ideate alternative practices for reporting and “intensive and continuous information sharing.” The presence of conflicting subgoals (for example, solutions for managing G-forces, engineering versus human factors) required “negotiation between the parties to reach agreement” (Scott and Levitt 2017, p. 97).

Like open innovation, open organizing is not immune to failures (e.g., Dahlander and Piezunka 2020), exposing the adopters to frictions across internal and external knowledge development, sense of identity, and intellectual property protection (e.g., Chesbrough 2020). Considering the idiosyncratic nature of some of our findings (e.g., secret gardens), we know our research is just scratching the surface of leveraging open organizing in less stable contexts. Here we consider the four new practices we observed, their implications, and future research to understand the dynamics of open organizing in contexts with VUCA attributes.

### How practices in unstable contexts differ from practices in more stable ones

Our literature review highlighted four internal practices for open organizing. These span governance (e.g., Crowston and Howison 2005; Dahlander and Frederiksen 2012; Xu and Alexy 2019; Dionne and Carlile 2019; Giustiniano et al. 2019), identification (e.g., Langner and Seidel 2015), information sharing (e.g., Henkel 2006; Alexy et al. 2013; Puranam et al. 2014; Hautz et al. 2017), and work design (e.g., Puranam et al. 2014). These practices focus on organizations in stable contexts—situations where the work can be predefined with modular tasks (Brusoni and Prencipe 2006). This prior work leaves open the question of whether these practices are effective for open organizing in situations in less stable settings—cases where organizations take on extreme complexity challenges (e.g., reducing oceanic plastic Porter et al. 2020).

At HyperloopTT, an organization built on the ideas of open organizing but also taking on the goal of transforming global mobility, we find significantly different practices than those found in open organizing for more stable contexts. In Table 3, we summarize these. While Clement and Puranam (2018, p. 3890) demonstrate via agent-based computational modeling that “the formal structure [may compensate] for the limits of decentralized efforts by agents”, we show that the emergence of practices can also compensate for the inner limitations of formal arrangements. New practices, microdynamics of work (Feldman and Orlikowski 2011), can emerge when contributors to open organizing encounter friction as they strive toward given and self-imposed goals.

**Table 3:** Comparison of practices for stable versus VUCA contexts

Stable	VUCA responses
**Reporting via a stable structure** Onion-shaped governance models of accountabilities and responsibilities: inner core holds the highest privileges for problem formulation and decision-making (e.g., Crowston and Howison 2005; Dahlander and Frederiksen 2012; Xu and Alexy 2019; Dionne and Carlile 2019; Giustiniano et al. 2019). Participation generally follows an apprenticeship model (Lave and Wenger 1991): newcomers typically start at the periphery and move inwards toward a core of decision-makers by showing commitment and capability	**Reporting via a dynamic porous structure** Reporting structure is problem-driven. Structural boundaries based on degree of interdependence. Participants manage a ‘functional’ onion structure to improve participant engagement and move across layers as the organization and their own needs change
**Identifying with an internally focused, internally bounded organizational identity** Strong identities provide clarity for boundaries around the community—who is in and who is out (Faraj et al. 2011). Culture encourages identity with the organization’s ideology (Raymond 1999; Stewart and Gosain 2006) and internal social ties (Langner and Seidel 2015). The organization attracts newcomers aligned with the identity (Wenger 1999)	**Identifying with an external movement that prioritizes societal change with little regard for organizational boundaries** Focus on a movement rather than limited to organizational boundaries. Identity evolves outside the organization and allows the organizational boundaries to blur with those of external partners and ecosystems. Boundaries tight enough to let contributors know they are inside, but not so tight as to limit effective action for the broader movement
**Sharing information openly** Transparent information sharing supporting workflow without hands-on management (Crowston and Howison 2005). Information transparency channels participant engagement to prioritized work (Faraj et al. 2011). Mechanisms include FAQs, discussion forums, voting, etc.	**Designing information sharing** Adaptable information sharing practices. Self-organization of evolving practices to enable innovative work while protecting valuable IP: triangulated permissioning (evaluation metric for determining whether sharing is possible) and secret gardens (closing aspects of work off from view so that other work can continue without demotivation or constraints of synchronization)
**Joining to predefined tasks** Practices for newcomers to match themselves to predefined tasks (Faraj et al. 2011). Practices engage and create commitment while maintaining competency requirements (von Krogh et al. 2003)	**Leaving work intentionally undefined** Work defined by engagement and allowing for different and changing availability. Work sharing, freedom to experiment, and the ability to take the next step rather than take on a defined task

*Practice 1: reporting via a dynamic porous structure* Prior work (e.g., Crowston and Howison 2005; Dahlander and Frederiksen 2012; Xu and Alexy 2019; Dionne and Carlile 2019) describes onion-shaped governance models with assigned layers for accountabilities and responsibilities. In those examples, set layers are important for shaping social interaction (Crowston and Howison 2005) and creating boundaries between those who are in the know and those who are not (Grey and Costas 2016). At HyperloopTT, we see instead a dynamic internal reporting structure crafted by unplanned interdependencies between tasks and contributors—rather than firm layers seen as boundaries for closure designed by the core team.

Structure was not about the static recognition of being in the know or the assignment of decision-making privileges, but rather in the possibility and encouragement to move across such boundaries. In sum, the porousness allowed for movement between circles, while the existence of the circles allowed for clarity about levels of engagement, transparency of information, and accountabilities (Langner and Seidel 2015; Giustiniano et al. 2019).

*Practice 2: identifying with an external movement that prioritizes societal change with little regard for organizational boundaries* The literature documents the role of strong, stable ideological ties with a project or a larger community (e.g., Stewart and Gosain 2006; Langner and Seidel 2015). We expand upon the value of strong ideological ties to suggest that identification can transcend a bond to the organization itself, supporting a bond to a higher-order change. By supporting identification with an external movement, the organization loses control over its ideology since it cannot control the broader outcomes. Focused on an external mission, the organization cannot specify that the outcome will be an encyclopedia, software, Lego designs, t-shirts, or an electric car. However, by losing control over the identification, the organization may gain the passionate, innovative participation it seeks. By being able to form the future, bold minds may come. Identification with an external movement allows the organization to blur its boundaries with external partners and the ‘world’, encouraging openness, creativity, innovation, and societal change. This broad identification also supports internal engagement. Only those who recognize the value of such change belong. Some contributors to HyperloopTT, such as the founders themselves, defined this identification to include competitors, welcoming every breakthrough by competitors as another step toward transforming the industry.

*Practice 3: designing information sharing* Evidence from other open organizations suggests transparency as a key to engage participants (Stewart and Gosain 2006; Faraj et al. 2011; Howison and Crowston 2014). Again, the organizations providing the background for those findings were in relatively stable contexts. In an organization dealing with a less stable context, we find participants defining practices managing openness for innovation while creating protection for intellectual property and avoiding innovation roadblocks. Triangulated permissioning promotes open information-sharing when more than one uninvolved person knows about the information and closure otherwise. Secret gardens release other teams’ innovation processes from the constraints of synchronization by hiding the existence of a team working on related projects. This is different, for example, from Shaikh and Vaast’s (2016) ‘digital folds’, where developers create private pockets of work and communication for efficiency. Secret gardens are a form of closure that allows open work to continue outside the garden without the risk of demotivating those excluded.

*Practice 4: leaving work intentionally undefined* Practices for matching work with available competencies are structurally necessary to any open organization (e.g., Puranam et al. 2014). Usually, such tasks are rigidly predefined when the ‘what’ is known in advance (e.g., contributions to Lego Ideas or Enel’s challenges, Di Fiore and Rosani 2021; El Sawy et al. 2016, respectively). Specific task separations presume that the discovery and explorative experimental process is sufficiently finalized (Raveendran et al. 2020). This is a dangerous assumption when the industry’s progress proceeds unpredictably (e.g., ConsenSys pitches, Majchrzak and Griffith 2020) or is excessively complex (e.g., Tuertscher et al. 2014; Porter et al. 2020). The case of HyperloopTT shows that a more emergent work design practice can accommodate exigencies in terms of what needs to be done and designed (Brusoni and Prencipe 2006). Sprints were ad hoc rather than defined. A unique go-between buddy system managed needs for technical expertise and administration. Focusing on a next step rather than a specific task goal allowed expertise to flow with individual availability. Energy for participation was used as a signal of project priority. These work design practices foster engagement as they enable those with little time to use their expertise in meaningful ways—in this case, managing a time–demand–skill triad.

### Novelty in the domain of organization design^3^

As pointed out by Burton et al. (2020, p. 1), “the field of organization design is undergoing a renaissance” in which many atypical forms flourish—i.e., the Organization Zoo (Puranam and Håkonsson 2015). It is, therefore, plausible to see elements of resemblance in such a vivid landscape where organizations are exposed to conflicting contingencies. For example, reporting via a dynamic porous structure (Practice 1) and the focus on designing information sharing (Practice 3) can also be found in holacracies (e.g., see Zappos, Robertson 2015; Moffett et al. 2021). Holacracies are organizational forms that try to replace the ‘implicit political power’ infused in traditional hierarchy-based structures. Nonetheless, as pointed out also by Bernstein et al. (2016), while holacracies strive to abolish the (dis-)functioning of traditional hierarchy, holacracies are not immune from the burden of formalization and clarity (in roles and rules), which translates into significant overhead.

Furthermore, holacracies require clear (and therefore formalized) structures, unlike other forms of flat and agile organizations. Unlike agile solutions (see, for example, Valve, Puranam and Håkonsson 2015), holacracies have clear rules for assigning to ‘circles’ and specific roles for gatewaying employee participation. Additionally, agile solutions see physical spaces and/or corporate values as central to organizational work, so they appear to work where the organizational boundary is well defined. HyperloopTT applies agility on a global scale and through a porous multiple-boundary structure.

Identifying with an external movement that prioritizes societal change (Practice 1) is also not new to the world (see also, Navis and Glynn 2011; Diriker et al. 2022). The unique blend of ideals and fierce competition makes the case of HyperloopTT distinctive, but at the same time extensible to similar situations. Such organizations are not focused purely on profit generation but rather—in the case of the hyperloop industry—the reinvention of an industry with global-scale benefits (e.g., see the outer layer “World” in the onion structure reported in Table 2).

Leaving work intentionally undefined (Practice 4) has conceptual roots in the garbage can model (Cohen et al. 1972). Nonetheless, as pointed out by von Hippel and von Krogh (2016, p. 217), the garbage can model requires “already formulated problems and solutions [which] are put into a ‘garbage can’ and linked and acted upon via managerial decisions.” Where HyperloopTT touches upon VUCA settings, both problems and solutions are generated in the making via globally distributed crowd-based teams. The organization must rely on different types of facilitators to promote a form of equifinality which is “both planned and serendipitous” (Giustiniano et al. 2019, p. 286)—a form of guided emergent organizing.

### Limitations

The above findings suffer from limitations based on the uniqueness of the setting and methodology. First, HyperloopTT is still a start-up. Second, the qualitative approach we adopted for our data analysis relies on the richness of our data and the familiarity with the case, but is not immune from critique. Reay et al. (2019) offer a possible criticism related to the necessity of selecting some pieces of evidence over others. Acknowledging qualitative data's complex and holistic nature, we tried to mitigate these limitations by focusing on a specific timespan and matching the observations to practices with attention in prior research. Finally, two of the authors being HyperloopTT contributors may trigger concerns regarding bias in the data. We hope that the level of transparency offered here and the deliberate choice of the composition of the research team alleviate those concerns, let the data speak for itself, and allow readers to follow the organization’s actions. Keeping these concerns in mind, we offer the following perspectives and next steps.

### Open organizing: a need to match to context

We offer a foundation to consider multi-layered practice-based approaches to open organizing. Organizational contexts vary and, especially in large organizations, there may be a range of instability within the same organization.

Others (e.g., Diriker et al. 2022) offer strategies for managing organizational openness with “punctuated” moments of closure to accommodate multi-stakeholder wicked problems. However, we suggest that level and location of stability privilege different practices. Additionally, different practices may motivate different people to engage with particular organizations. Moreover, while some scholars suggest that open organizing requires clear tasks to make it simple for the public to join (e.g., von Krogh et al. 2003), our findings suggest that task clarity is not necessary for open organizing. Consequently, a firm’s policy for implementing open organizing does not have to reduce participants to task workers. Instead, designers must match the practice to the firm's stability, division, or product context.

We acknowledge that much research is still needed and that our four practices are not one-size-fits-all solutions nor perfect. HyperloopTT’s examples often contain tradeoffs that might or might not work in another setting. We believe a deeper analysis of the contingencies for which those practices represent a fit is required. In particular: types of open innovation (e.g., inside-out, outside-in, Chesbrough and Appleyard 2007), level of guided emergence of organization design and intensity of the crowd engagement (crowd-open vs. crowd-based organizing, Giustiniano et al. 2019), types of interdependence (Raveendran et al. 2020), and selectivity in openness and closedness (Dobusch et al. 2019). Research on multi-layering different practice-based approaches to open organizing should also be examined—particularly in large organizations where the stability participants encounter may vary across the organization.

### Open organizing practices support a new class of workers: creative work designers rather than task workers

Beyond a deeper understanding of the role of context, the four practices also call for a new kind of work and workers. The porousness of the boundaries requires the capacity to activate a spirit of initiative leveraging engagement, transparency of information, and accountabilities. The sense of affiliation to a movement (e.g., reinventing transportation) must be more robust than in other forms of open organizing (e.g., bring your ideas). Affiliation is essential to organizational engagement, including welcoming competitors if they contribute to the mission. (By contrast, it’s more challenging to imagine Lego designers being happy with Mattel’s progress). Open organizations in stable contexts allow participants to generally benefit from open information and knowledge, secreting away what the focal organization believes to undisclosable (see Dobusch et al. 2019 on open strategy at Wikimedia). When information is—paradoxically—both open and closed, participants get familiar with the idea that not everything visible is accessible and not everything confidential is not accessible.

When work is intentionally undefined, or at least undefined for as long as possible, organizations’ flexibility surpasses even the most advanced adoption of agile organizing (see Spotify).^4^ We saw the practices of open organizing enabling participants to engage in exploration and experimentation related both to products, services, and the design of the work itself. This is different and more open-ended than, for example, internally or externally focused hackathons (e.g., Pe-Than et al. 2022) more focused on point solutions. The ad hoc definition of work allows individuals to blend their expertise and availability (see also Burton et al. 2017). These features mark the differences between participants aligning to defined and un-defined tasks and missions. Crowd contributors in HyperloopTT did not participate in the way expected from research on organizations in more stable contexts.

*Creative work designers* We call out the behaviors at HyperloopTT as signaling a new worker classification: creative work designers. Past research suggesting that workers craft the design of their jobs has generally focused on employees (Lazazzara et al. 2020; Raveendran et al. 2020). Though we can draw insights from project-based organizations (e.g., project workers taken from competence pools and temporarily assigned to projects, Bredin and Söderlund 2011) and labor-market intermediaries, the literature says very little about work design by part-time, volunteer, public participants.

*We suggest that engaging participants in open organizing when the context has VUCA attributes requires contributors participate in a creative work design process.*

Rather than a focus on reducing the work to modularized tasks and encouraging participants to be task workers (e.g., Crowston and Howison 2005; Dahlander and Frederiksen 2012; Puranam et al. 2014; El Sawy et al. 2016; Xu and Alexy 2019; Dionne and Carlile 2019), practices for VUCA contexts instead encourage creative work design. Here, crowd contributors who deal with ambiguous tasks boast energy and ambidexterity that embeds technical competencies and organization design skills. The mechanisms are in line with recent research on hackathon teams. Lifshitz-Assaf et al. (2021) find teams that maintain temporal ambiguity and interlink their process with evolving coordination can accelerate innovation—both in having ideas and following through on those ideas. We see the success of HyperloopTT and the data we have gathered as supporting an open organization/open innovation approach that engages participants at the design level for creative work.

*Meso link to organizational design* Our observations link individual and top-down work design practices (e.g., Parker et al. 2017) to organizational design and practice. Raveendran et al. (2020) note the value bottom-up design offers in dynamic work streams. Open organizing in VUCA contexts may intensify this value. Job crafting (e.g., Wrzesniewski and Dutton 2001; Tims et al. 2015) describes occasions where people independently adjust their work's task, cognitive, and relational boundaries. Achievement, meaningfulness, and satisfaction are all motivators (e.g., Zhang and Parker 2019). Work crafting (using ‘work’ rather than ‘job’ given the varieties of work arrangements in the open organizing context) likely benefits task workers and creative work designers. However, in contexts with VUCA attributes, it may be imperative that the creative work designer role is explicit. This may be a fruitful area to extend from Tuertscher et al. (2014) and Porter et al. (2020) examinations of communities supporting grand initiatives.

*Furthering knowledge practices* Tuertscher et al. (2014) offer a blended set of practices for knowledge sharing critical for developing the CERN ATLAS particle detector. Cycles of knowledge contestation and justification result in interlaced/shared knowledge that, along with boundary infrastructures, support coordination and project success in their complex context. We would describe the ATLAS scientists and engineers as creative work designers. Roles at CERN were fluid, and the higher-level coordination mechanism was emergent. HyperloopTT’s practices offer more fine-grained examples of individually practiced creative work design. Additionally, the HyperloopTT practices are an expansion as the infrastructure boundaries at HyperloopTT are less rigid, given most of the contributors at the time of our research were individuals and not working on behalf of partner organizations.

*Furthering robust action* Porter et al. (2020) scale the principles of robust action (participatory architecture, coordination through multivocal inscriptions, and progress through distributed experimentation, Ferraro et al. 2015). Focused on saving our oceans, they find that, beyond “generating novelty and sustaining engagement, robust action must also generate engagement and sustain novelty” (Porter et al. 2020 p. 276). Their context is complex and uncertain (as a grand challenge), sometimes volatile (participants turn over), and ambiguous, given the variety of meanings that can be ascribed to crowdsourced ideas. Yet, they identify microdynamics to engage new participants and engender ongoing commitment to novel solutions identified by prior participants. The practices at HyperloopTT may offer additional insights into these and other microdynamics supporting robust action. Creative work design keeps open options for additional microdynamics across different stages of work that may confront VUCA contexts.

*Broader issues* By expanding the practices of open organizing, we can also support competitive advantage in settings where open organizing is less effective: nascent markets and gig work. For example, Bremner and Eisenhardt (2021) find that the firm organizing form, given its defined coordination mechanisms, is superior to a community form when working in nascent markets. The HyperloopTT example suggests context stability as an additional dimension.

We can also consider these results beyond a single organization setting. Current approaches to gig work focus on definable tasks (Webster 2016). However, creative work design might support gig work in settings with unstructured tasks. The gig may be connected to time rather than task. From production to management, this new form of gig work and gig workers could support organizations with volatile, uncertain, complex, or ambiguous contexts rather than a race-to-the-bottom to micro-tasks and limited compensation (e.g., Deng et al. 2016).

We also wonder whether responses to VUCA environments will follow different trajectories over time. Kane and Ransbotham (2016), for example, show decreased popularity in participation in Wikipedia and OSS communities, respectively. A shift to less reductive approaches could curb this trend.

Seeing differences between task workers and creative work designers generates further avenues for future research. What are the boundary conditions for creative work designers developing solutions? In terms of participants’ commitment and engagement, are the forms of open organizing sustainable over time when the persistence of instability may generate stress, or the extreme complexity may return to chaos? Can the organization expect public crowds to react in an aligned way to the solicitations triggered by its organizational practices when the ambition is to accomplish something difficult to specify (c.f., goal setting, Locke and Latham 1984)? Will task workers become jealous of creative work designers? Should the incentives be different? Can workers transition through different practices when the organization’s needs change? These are questions to address as multiple forms of open organizing leverage open innovation and permeate organizational designs.

## Conclusion

We observed Hyperloop Transportation Technologies (HyperloopTT). HyperloopTT is unique in that it competes with traditional organizations, generates valuable intellectual property, yet uses a foundational workforce of global part-time contributors, not employees. This open organization aims to transform global mobility while functioning in a context with volatile, uncertain, complex, and ambiguous attributes (VUCA). The complexity, interdisciplinarity, and extreme societal interdependence preclude applying the standard open organizing practices of task reduction. Our 2-year observation identifies four practices not found in more typical, predefined, open organizing. Despite the resemblance of some aspects of the identified practices to models and forms available in the literature, in HyperloopTT we see a confluence of practices that appear unique as a form of “design of emergence” (Eisenman et al. 2020, p. 1). We expand perspectives on open organizing to consider aspects of VUCA versus stable contexts and the possibilities of shifting the roles of open organizing participants from task workers to creative work designers. Applications of these findings may give more contexts access to effective open organizing and bring even greater creativity to their work and functioning.

## Data Availability

Due to the competitive nature of the industry, we cannot share the data.

## Footnotes

1 HyperloopTT is a leading company in the hyperloop industry, developing a new form of transportation intended to disrupt all current forms of mass transportation. HyperloopTT needs highly specialized engineering and scientific experts for the innovations required as they develop a functioning hyperloop. This complex innovation system has many engineering and scientific challenges (Dougherty, 2017). These include: designing for low cost and sustainability, minimizing passengers’ adverse reactions to G-forces, efficient levitation and braking, ‘last mile’ complexities, resolving Kantowitz limits (a challenge in the physics of hyperloops), building and maintaining hundreds of miles of vacuum chambers, and creating regulatory and insurance standards for a mode of transportation that is neither rail nor air. There are currently five main competitors in the industry, with only HyperloopTT demonstrating a full-scale working prototype. HyperloopTT works with partner organizations as well as individual contributors. Majchrzak et al. (2018) describe HyperloopTT as a catalyst organization, using “…an almost unlimited availability of specialized knowledge to create and capture an almost unlimited supply of unanticipatable opportunities” (p. 474). As noted earlier, the data here are an expansion of those described in Majchrzak et al. (2018) and are considered with filters more appropriate for internal practice.

2 We became formal contributors, signing the same non-disclosure agreement (NDA) that all contributors sign and receiving a standard contract providing stock options in exchange for work accomplished. HyperloopTT designated us Senior Operations Managers, reporting to the Operations Director with the expectation we would contribute 10 h a week. In exchange, we obtained permission to become participant observers with access to all contributors, all reports and slide decks, and all information shared on the collaborative platform, Facebook’s Workplace. To minimize conflict of interest, we only entered hours into the stock accounting system for the few tasks unrelated to the research observations.

3 We are grateful to one of the anonymous reviewers for encouraging us to elaborate on these topics and for their thoughtful guidance.

4 Spotify notes that they’ve had to move their development strategies from SCRUM to a custom form of Agile to be as responsive as they need. https://www.youtube.com/watch?v=4GK1NDTWbkY HyperloopTT’s design seems still more responsive.
